# Relationship Between Parental Lifestyle and Dietary Habits of Children: A Cross-Sectional Study

**DOI:** 10.2188/jea.JE20190015

**Published:** 2020-06-05

**Authors:** Kumi Tenjin, Michikazu Sekine, Masaaki Yamada, Takashi Tatsuse

**Affiliations:** Department of Epidemiology and Health Policy, Graduate School of Medicine and Pharmaceutical Sciences, University of Toyama, Toyama, Japan

**Keywords:** nutrition education, dietary habit, lifestyle factors

## Abstract

**Background:**

Although parents seek the best for their children, nutrition education for parents has attracted little attention to improve their children’s dietary habits. To address this gap, this study aimed to examine the relationship between parental lifestyle factors and children’s dietary habits.

**Methods:**

We used data from the questionnaire survey of the Super Shokuiku School Project conducted in January 2016. The participants consisted of 1,632 elementary school children who answered questions about their lifestyle, while their parents answered parental lifestyle questions, including Breslow’s seven health practice score (BHPS). Logistic regression analysis was performed to evaluate the strength of the relationship between parental lifestyle factors and parental dietary attitudes or children’s dietary habits.

**Results:**

Compared with good maternal BHPS (scores of 6–7), poor maternal BHPS (scores of 0–3) was significantly associated with less parental interest in Shokuiku, less parental consideration of nutrient balance, and an increased rate of children eating breakfast alone (adjusted odds ratio [aOR] 2.95; 95% confidence interval [CI], 1.82–4.78, aOR 3.86; 95% CI, 2.50–5.96, and aOR 2.42; 95% CI, 1.34–4.35, respectively). There was no significant difference between parental BHPS and the following children’s dietary habits: frequency of eating breakfast, vegetable intake, and snacking. These habits of children were associated with their personal lifestyle factors.

**Conclusion:**

Two types of dietary habits among children were associated with lifestyle factors of both parents and children. Nutrition education might be especially important for parents to improve their dietary attitude and children’s dietary habits. However, different nutrition education interventions would be needed to appropriately address each dietary habit.

## INTRODUCTION

Many previous studies have shown that children’s lifestyle factors correlate with their physical and psychological health. For instance, childhood obesity is associated with lifestyle factors, such as lack of sleep, excess screen time, skipping breakfast, and a lower frequency of family meals.^1^^–^^5^ Furthermore, undesirable lifestyle factors, especially with respect to dietary habits, are possible risk factors for constipation, feelings regarding school avoidance, and poor quality of life (QOL).^6^^–^^8^ Therefore, improving dietary habits via nutrition education is important for promoting the health of children. In 2005, Japan enacted the Basic Law on Shokuiku, the first law to promote healthy diets and eating habits among the Japanese population.^9^ This law defines “*Shokuiku*” as the acquisition of knowledge about food and nutrition, as well as the ability to make appropriate food choices through various experiences related to food; these elements combine to develop people’s ability to practice a healthy diet.^10^

Although many schools in Japan offer nutrition education to improve children’s dietary habits, little attention has been paid to the value of nutrition education for parents.^11^ Several researchers have focused on the relationship between dietary habits of children and their family environment. One study suggested that having enjoyable eating experiences at home during childhood promoted healthy dietary habits and good QOL later in life.^12^ Other studies have indicated that an undesirable family environment may exacerbate poor dietary habits among children and be associated with increased body mass index.^13^^,^^14^ Furthermore, a previous study investigating dietary ideals and realities showed that the most significant barrier to integrating dietary ideals and realities was work-life balance, especially in terms of lack of time and family life rhythm.^15^ We believe that family environment, including parental lifestyle factors, has a significant effect on the dietary habits of children. However, there has been little research investigating such relationships comprehensively. To effectively improve the quality of nutrition education, it is important to elucidate any such relationships.

Thus, the present study aimed to examine the relationship between parental lifestyle factors and dietary habits of elementary school children in Japan using data from the Ministry of Education, Culture, Sports, Science and Technology (MEXT) Super Shokuiku School Project.

## METHODS

### Participants

The MEXT Super Shokuiku School Project is a nutrition education project aimed at promoting healthy lifestyles in school children and improving their health via nutrition education.^7^^,^^16^ As part of this project, three questionnaire surveys have been conducted thus far. The initial questionnaire survey (phase I) was conducted in May 2014, before nutrition education, which taught the importance of eating a healthy diet through certain events (such as agricultural experiences), was offered. After conducting nutrition education, two more rounds of surveys were conducted: phase II took place in December 2014 and phase III in January 2016. These questionnaire surveys were intended for all children who attended five elementary schools in Takaoka city, Toyama Prefecture at that time. Because the present study aimed to examine the relationship between parental lifestyle factors and children’s dietary habits, we used findings obtained from the January 2016 phase III survey, which included data about parental lifestyle factors. The total population of the phase III survey consisted of 2,129 children who were in the first through sixth grades at the five elementary schools. Of those who received the questionnaire, 1,986 children responded, for a response rate of 93.3%. Respondents were excluded from the analysis if they did not answer one or more questions relevant to the present study. The remaining 1,632 children comprise the present study’s participant populations.

The survey was approved by the institutional review board of the University of Toyama. Both children and their parents provided written informed consent and participated in the survey voluntarily.

### Questionnaire

There were two sections in the questionnaire: one for children and one for parents. In the section for children, children answered the questionnaire under parental supervision. The items in the section for parents were filled in only by the parents.

Children reported on their sex, grade, dietary habits, and lifestyle factors by choosing the most appropriate answer from several options. Grade was categorized into six groups ranging from first to sixth grade. Dietary habits included the frequency of eating breakfast, the person with whom the child ate breakfast, the frequency of vegetable intake, and the frequency of snacking. Responses for each were dichotomized on the basis of whether the habit was desirable or undesirable, as follows: (1) the frequency of eating breakfast: “breakfast skipping” and “non-breakfast skipping”, in which breakfast skipping was defined as not eating breakfast every day; (2) the person with whom the child ate breakfast: “alone” and “with someone”; (3) the frequency of vegetable intake: “always or often” and “sometimes or almost never”; and (4) the frequency of snacking: “less than twice a day” and “twice a day or more”. Lifestyle factors included sleeping hours, screen time, and physical activity. These responses were each categorized into three groups that included the most frequent answer, and the one directly lower and higher than it: (1) sleeping hours: less than 8 hours, 8 to 9 hours, or 9 hours or more; (2) screen time: less than 1 hour, 1 to 2 hours, or 2 hours or more; and (3) physical activity: very often, often, or rarely or almost never. Previous studies have validated the lifestyle questionnaire in terms of sleeping hours and physical activity.^17^^,^^18^

Parents responded to the following items: dietary attitudes, family environment, and their own lifestyle factors by choosing the most appropriate answer from several options. Dietary attitudes included the parental interest in Shokuiku and their consideration of nutrient balance. Responses were dichotomized on the basis of whether the response was positive or negative: (1) interest in Shokuiku: “yes” and “no or do not know”; and (2) consideration of nutrient balance: “yes” and “rarely or no”. Family environments included family members, maternal employment status, and family affluence. These responses were also dichotomized on the basis of whether the response was positive or negative: (1) family members with whom children live: “only parent(s)” and “not only parent(s)”; (2) maternal employment status: “employed” and “not employed”; and (3) family affluence: “affluent” and “not affluent,” in which parents who answered neither affluent nor not affluent were included in the affluent group.

Lifestyle factors were assessed by Breslow’s seven health practice score (BHPS). BHPS is widely used to assess lifestyle variables in Japan and other countries, and an inverse relationship between BHPS and age-adjusted mortality has been confirmed.^19^^–^^22^ The BHPS measures respondents’ adherence to seven good health practices: never smoking cigarettes, engaging in regular physical activity, drinking alcohol in moderation or not at all, getting 7 to 8 hours sleep regularly, maintaining proper weight, eating breakfast, and not eating between meals. Parents answered yes or no to these seven items. “Yes” responses were summed to provide a cumulative BHPS ranging from 0 to 7, which we categorized into three groups: poor (0–3), moderate (4–5), and good (6–7), just as in the original study.^22^

### Statistical analysis

We used logistic regression analysis to evaluate the strength of the relationship between parental lifestyle factors and parental dietary attitudes or dietary habits of children. To control for potential confounding factors for the relationship between parental lifestyle factors and dietary attitudes, we performed a multivariate analysis adjusting for family members, maternal employment status, and family affluence as well as a univariate analysis. We also performed a multivariate analysis to evaluate the relationship between parental lifestyle factors and children’s dietary habits, adjusting for sex, grade, sleeping hours, screen time, physical activity, family members, maternal employment status, and family affluence. Correlations and multicollinearity between independent variables were examined using Spearman rank correlation coefficients and variance inflation factors, respectively. The threshold for significance was two-tailed *P* values of less than 0.05. All statistical analyses were conducted using IBM SPSS version 20.0 (IBM Corp, Armonk, NY, USA).

## RESULTS

The characteristics of the participants are presented in Table 1. There was little difference in the distribution of sex and grade, respectively. Regarding children’s lifestyle factors, the most frequent answers to the questions assessing sleeping hours, screen time, and physical activity were 8–9 hours, 1–2 hours, and often, respectively. When examining the lifestyle factors of parents, the moderate scoring band was the most frequent for both paternal and maternal BHPS. Parents with poor BHPS were more likely to answer “not affluent” to the question assessing family affluence (data not shown).

**Table 1.** Characteristics of the participants

	Total (*n* = 1,632)
	*n* (%)
Sex, female	818 (50.1)
Grade	
1	278 (17.0)
2	271 (16.6)
3	271 (16.6)
4	240 (14.7)
5	282 (17.3)
6	290 (17.8)
Sleeping hours, hours	
<8	340 (20.8)
8–9	942 (57.7)
≥9	350 (21.4)
Screen time, hours	
<1	258 (15.8)
1–2	742 (45.5)
≥2	632 (38.7)
Physical activity	
Very often	455 (27.9)
Often	732 (44.9)
Rarely or almost never	445 (27.3)
Family members, only parent(s)	1074 (65.8)
Maternal employment status, employed	1391 (85.2)
Family affluence, not affluent	406 (24.9)
Paternal BHPS	
Good (6–7)	356 (21.8)
Moderate (4–5)	711 (43.6)
Poor (0–3)	565 (34.6)
Maternal BHPS	
Good (6–7)	445 (27.3)
Moderate (4–5)	894 (54.8)
Poor (0–3)	293 (18.0)

BHPS, Breslow’s seven health practice score.

Table 2 shows the relationship between parental lifestyle factors and dietary attitudes. Parents in non-affluent families were less likely to state an interest in Shokuiku. Poor maternal BHPS was significantly associated with less interest in Shokuiku compared with good maternal BHPS (adjusted odds ratio [OR] 2.95; 95% confidence interval [CI], 1.82–4.78). Parents in non-affluent families were less likely to consider nutrient balance when making dietary choices. When compared to good maternal BHPS, mothers who scored moderate or poor for BHPS were significantly less likely to consider nutrient balance (adjusted OR 1.85; 95% CI, 1.26–2.71 and adjusted OR 3.86; 95% CI, 2.50–5.96, respectively).

**Table 2.** The relationship between parental lifestyle factors and dietary attitudes

	Interest in Shokuiku			Consideration of nutrient balance		
	
	No or do not know *n* (%)	Univariate OR (95% CI)	Multivariate OR (95% CI)	Rarely or no *n* (%)	Univariate OR (95% CI)	Multivariate OR (95% CI)
Family members						
Only parent(s)	121 (11.3)	0.93 (0.68–1.28)	0.88 (0.64–1.22)	192 (17.9)	1.15 (0.87–1.51)	1.08 (0.82–1.44)
Not only Parent(s)	67 (12.0)	1.00	1.00	89 (15.9)	1.00	1.00
Maternal employment status						
Employed	161 (11.6)	1.04 (0.67–1.60)	0.97 (0.63–1.51)	240 (17.3)	1.02 (0.71–1.46)	0.97 (0.66–1.41)
Not employed	27 (11.2)	1.00	1.00	41 (17.0)	1.00	1.00
Family affluence						
Affluent	124 (10.1)	1.00	1.00	185 (15.1)	1.00	1.00
Not affluent	64 (15.8)	1.66 (1.20–2.30)^*^	1.46 (1.05–2.04)^*^	96 (23.6)	1.74 (1.32–2.30)^*^	1.52 (1.14–2.03)^*^
Paternal BHPS						
Good (6–7)	27 (7.6)	1.00	1.00	45 (12.6)	1.00	1.00
Moderate (4–5)	75 (10.5)	1.44 (0.91–2.28)	1.23 (0.77–1.97)	97 (13.6)	1.09 (0.75–1.59)	0.89 (0.60–1.32)
Poor (0–3)	86 (15.2)	2.19 (1.39–3.45)^*^	1.53 (0.94–2.48)	139 (24.6)	2.26 (1.56–3.25)^*^	1.47 (0.99–2.17)
Maternal BHPS						
Good (6–7)	32 (7.2)	1.00	1.00	39 (8.8)	1.00	1.00
Moderate (4–5)	92 (10.3)	1.48 (0.97–2.25)	1.33 (0.86–2.04)	148 (16.6)	2.07 (1.42–3.00)^*^	1.85 (1.26–2.71)^*^
Poor (0–3)	64 (21.8)	3.61 (2.29–5.68)^*^	2.95 (1.82–4.78)^*^	94 (32.1)	4.92 (3.26–7.41)^*^	3.86 (2.50–5.96)^*^

BHPS, Breslow’s seven health practice score; CI, confidence interval; OR, odds ratio.

In multivariate model, all of the variables were simultaneously entered.

^*^P < 0.05.

Table 3 shows the relationship between parental lifestyle factors and breakfast habits of children. Breakfast skipping behavior was significantly associated with the following factors: female gender, short sleeping hours, and long screen time. There was no significant association between parental BHPS and breakfast skipping. Children who lived with only their parents were more likely to eat breakfast alone than who lived with others as well. With reference to good maternal BHPS, moderate and poor maternal BHPS were significantly associated with children who ate breakfast alone (adjusted OR 1.87; 95% CI, 1.13–3.11 and adjusted OR 2.42; 95% CI, 1.34–4.35, respectively).

**Table 3.** The relationship between parental lifestyle factors and breakfast habits of children

	Frequency of eating breakfast			Person with whom children eat breakfast		
	
	Breakfast skipping *n* (%)	Univariate OR (95% CI)	Multivariate OR (95% CI)	Alone *n* (%)	Univariate OR (95% CI)	Multivariate OR (95% CI)
Sex						
Male	43 (5.3)	1.00	1.00	77 (9.5)	1.00	1.00
Female	61 (7.5)	1.44 (0.97–2.16)	1.54 (1.01–2.34)^*^	72 (8.8)	0.92 (0.66–1.29)	0.96 (0.68–1.36)
Grade						
1	14 (5.0)	1.00	1.00	24 (8.6)	1.00	1.00
2	10 (3.7)	0.72 (0.32–1.66)	0.63 (0.27–1.46)	25 (9.2)	1.08 (0.60–1.93)	1.13 (0.62–2.07)
3	15 (5.5)	1.10 (0.52–2.34)	0.90 (0.42–1.95)	19 (7.0)	0.80 (0.43–1.49)	0.78 (0.41–1.49)
4	18 (7.5)	1.53 (0.74–3.14)	1.16 (0.55–2.47)	18 (7.5)	0.86 (0.45–1.62)	0.76 (0.39–1.49)
5	32 (11.3)	2.41 (1.26–4.63)^*^	1.82 (0.91–3.63)	25 (8.9)	1.03 (0.57–1.85)	0.96 (0.52–1.80)
6	15 (5.2)	1.03 (0.49–2.17)	0.75 (0.34–1.64)	38 (13.1)	1.60 (0.93–2.74)	1.35 (0.75–2.42)
Sleeping hours, hours						
<8	31 (9.1)	3.09 (1.53–6.26)^*^	2.30 (1.08–4.90)^*^	43 (12.6)	1.66 (1.01–2.75)^*^	1.39 (0.80–2.41)
8–9	62 (6.6)	2.17 (1.13–4.17)^*^	1.71 (0.86–3.39)	78 (8.3)	1.04 (0.66–1.63)	0.95 (0.59–1.53)
≥9	11 (3.1)	1.00	1.00	28 (8.0)	1.00	1.00
Screen time, hours						
<1	9 (3.5)	1.00	1.00	17 (6.6)	1.00	1.00
1–2	35 (4.7)	1.37 (0.65–2.89)	1.44 (0.67–3.09)	50 (6.7)	1.02 (0.58–1.81)	0.95 (0.53–1.70)
≥2	60 (9.5)	2.90 (1.42–5.94)^*^	2.75 (1.30–5.82)^*^	82 (13.0)	2.11 (1.23–3.64)^*^	1.75 (0.99–3.09)
Physical activity						
Very often	30 (6.6)	1.00	1.00	41 (9.0)	1.00	1.00
Often	45 (6.1)	0.93 (0.58–1.50)	0.89 (0.54–1.46)	55 (7.5)	0.82 (0.54–1.25)	0.80 (0.52–1.23)
Rarely or almost never	29 (6.5)	0.99 (0.58–1.67)	0.77 (0.44–1.34)	53 (11.9)	1.37 (0.89–2.10)	1.14 (0.73–1.79)
Family members						
Only parent(s)	71 (6.6)	1.13 (0.74–1.72)	1.15 (0.74–1.79)	111 (10.3)	1.58 (1.07–2.31)^*^	1.64 (1.11–2.43)^*^
Not only parent(s)	33 (5.9)	1.00	1.00	38 (6.8)	1.00	1.00
Maternal employment status						
Employed	95 (6.8)	1.89 (0.94–3.80)	1.58 (0.77–3.23)	125 (9.0)	0.89 (0.56–1.41)	0.92 (0.57–1.48)
Not employed	9 (3.7)	1.00	1.00	24 (10.0)	1.00	1.00
Family affluence						
Affluent	73 (6.0)	1.00	1.00	110 (9.0)	1.00	1.00
Not affluent	31 (7.6)	1.31 (0.84–2.02)	1.12 (0.71–1.76)	39 (9.6)	1.08 (0.73–1.58)	0.92 (0.62–1.37)
Paternal BHPS						
Good (6–7)	17 (4.8)	1.00	1.00	21 (5.9)	1.00	1.00
Moderate (4–5)	26 (3.7)	0.76 (0.41–1.41)	0.67 (0.35–1.27)	62 (8.7)	1.52 (0.91–2.54)	1.31 (0.77–2.22)
Poor (0–3)	61 (10.8)	2.41 (1.39–4.20)^*^	1.78 (0.97–3.26)	66 (11.7)	2.11 (1.27–3.51)^*^	1.47 (0.85–2.54)
Maternal BHPS						
Good (6–7)	22 (4.9)	1.00	1.00	21 (4.7)	1.00	1.00
Moderate (4–5)	52 (5.8)	1.19 (0.71–1.98)	0.98 (0.57–1.68)	87 (9.7)	2.18 (1.33–3.56)^*^	1.87 (1.13–3.11)^*^
Poor (0–3)	30 (10.2)	2.19 (1.24–3.88)^*^	1.26 (0.67–2.37)	41 (14.0)	3.28 (1.90–5.69)^*^	2.42 (1.34–4.35)^*^

BHPS, Breslow’s seven health practice score; CI, confidence interval; OR, odds ratio.

In multivariate model, all of the variables were simultaneously entered.

^*^P < 0.05.

Table 4 shows the relationship between parental lifestyle factors and children’s frequency of vegetable intake or snacking. Older children and children who lived with only their parents were more likely to eat vegetables frequently. Meanwhile, long screen time, physical inactivity, and coming from a non-affluent family were significantly associated with lower vegetable intake. Longer screen time was significantly associated with high frequency of snacking. There was no significant association between parental BHPS and the frequency of these dietary habits among children.

**Table 4.** The relationship between parental lifestyle factors and the frequency of vegetable intake or snacking of children

	Frequency of vegetable intake			Frequency of snacking		
	
	Sometimes or almost never *n* (%)	Univariate OR (95% CI)	Multivariate OR (95% CI)	twice a day or more *n* (%)	Univariate OR (95% CI)	Multivariate OR (95% CI)
Sex						
Male	169 (20.8)	1.00	1.00	100 (12.3)	1.00	1.00
Female	146 (17.8)	0.83 (0.65–1.06)	0.87 (0.67–1.12)	103 (12.6)	1.03 (0.77–1.38)	1.17 (0.87–1.59)
Grade						
1	59 (21.2)	1.00	1.00	35 (12.6)	1.00	1.00
2	56 (20.7)	0.97 (0.64–1.46)	0.89 (0.58–1.37)	34 (12.5)	1.00 (0.60–1.65)	0.92 (0.55–1.55)
3	49 (18.1)	0.82 (0.54–1.25)	0.71 (0.45–1.10)	33 (12.2)	0.96 (0.58–1.60)	0.91 (0.54–1.54)
4	55 (22.9)	1.10 (0.73–1.67)	0.88 (0.57–1.37)	29 (12.1)	0.95 (0.56–1.61)	0.88 (0.51–1.53)
5	49 (17.4)	0.78 (0.51–1.19)	0.62 (0.40–0.98)^*^	32 (11.3)	0.89 (0.53–1.48)	0.82 (0.48–1.41)
6	47 (16.2)	0.72 (0.47–1.10)	0.53 (0.33–0.83)^*^	40 (13.8)	1.11 (0.68–1.81)	0.96 (0.57–1.62)
Sleeping hours, hours						
<8	72 (21.2)	1.27 (0.87–1.86)	1.32 (0.87–2.00)	49 (14.4)	1.14 (0.74–1.76)	1.05 (0.65–1.69)
8–9	182 (19.3)	1.13 (0.82–1.56)	1.11 (0.79–1.55)	109 (11.6)	0.89 (0.61–1.29)	0.84 (0.57–1.24)
≥9	61 (17.4)	1.00	1.00	45 (12.9)	1.00	1.00
Screen time, hours						
<1	25 (9.7)	1.00	1.00	13 (5.0)	1.00	1.00
1–2	131 (17.7)	2.00 (1.27–3.15)^*^	1.81 (1.14–2.87)^*^	85 (11.5)	2.44 (1.34–4.45)^*^	2.56 (1.39–4.70)^*^
≥2	159 (25.2)	3.13 (2.00–4.91)^*^	2.68 (1.68–4.28)^*^	105 (16.6)	3.75 (2.07–6.81)^*^	4.16 (2.26–7.68)^*^
Physical activity						
Very often	68 (14.9)	1.00	1.00	62 (13.6)	1.00	1.00
Often	134 (18.3)	1.28 (0.93–1.75)	1.25 (0.90–1.74)	93 (12.7)	0.92 (0.65–1.30)	0.86 (0.61–1.23)
Rarely or almost never	113 (25.4)	1.94 (1.39–2.71)^*^	1.84 (1.30–2.61)^*^	48 (10.8)	0.77 (0.51–1.15)	0.66 (0.44–1.00)
Family members						
Only parent(s)	183 (17.0)	0.66 (0.52–0.85)^*^	0.66 (0.51–0.86)^*^	141 (13.1)	1.21 (0.88–1.66)	1.29 (0.93–1.79)
Not only parent(s)	132 (23.7)	1.00	1.00	62 (11.1)	1.00	1.00
Maternal employment status						
Employed	274 (19.7)	1.20 (0.83–1.72)	1.18 (0.81–1.73)	178 (12.8)	1.27 (0.81–1.97)	1.27 (0.80–2.00)
Not employed	41 (17.0)	1.00	1.00	25 (10.4)	1.00	1.00
Family affluence						
Affluent	212 (17.3)	1.00	1.00	144 (11.7)	1.00	1.00
Not affluent	103 (25.4)	1.63 (1.24–2.13)^*^	1.52 (1.15–2.01)^*^	59 (14.5)	1.28 (0.92–1.77)	1.19 (0.85–1.67)
Paternal BHPS						
Good (6–7)	58 (16.3)	1.00	1.00	38 (10.7)	1.00	1.00
Moderate (4–5)	132 (18.6)	1.17 (0.83–1.64)	1.04 (0.73–1.48)	93 (13.1)	1.26 (0.84–1.88)	1.16 (0.77–1.76)
Poor (0–3)	125 (22.1)	1.46 (1.03–2.06)^*^	1.17 (0.81–1.71)	72 (12.7)	1.22 (0.81–1.86)	1.01 (0.64–1.59)
Maternal BHPS						
Good (6–7)	75 (16.9)	1.00	1.00	51 (11.5)	1.00	1.00
Moderate (4–5)	175 (19.6)	1.20 (0.89–1.62)	1.01 (0.74–1.39)	110 (12.3)	1.08 (0.76–1.54)	1.00 (0.69–1.45)
Poor (0–3)	65 (22.2)	1.41 (0.97–2.04)	1.00 (0.66–1.50)	42 (14.3)	1.29 (0.83–2.00)	1.07 (0.66–1.72)

BHPS, Breslow’s seven health practice score; CI, confidence interval; OR, odds ratio.

In multivariate model, all of the variables were simultaneously entered.

^*^P < 0.05.

The strength of the relationships between paternal BHPS and parental dietary attitudes or children’s dietary habits was attenuated in the multivariate analysis. The largest reduction of the association between paternal BHPS and parental dietary attitudes or children’s breakfast habits was observed when the analysis was adjusted for maternal BHPS. Meanwhile, children’s screen time most weakened the relationship between paternal BHPS and the frequency of vegetable intake (data not shown).

Spearman rank correlation coefficients between independent variables ranged from −0.35 to 0.33. The maximum of the variance inflation factor was 1.161. Multicollinearity was not confirmed in the multivariate analysis.

## DISCUSSION

The present study investigated the relationship between parental lifestyle and dietary habits of children using data from the MEXT Super Shokuiku School Project. We revealed that low maternal BHPS was dose-dependently associated with lack of parental concern about diet and children eating breakfast alone. Other notable dietary habits of children were significantly associated not with parental BHPS but with the children’s own lifestyle factors.

Previous studies showed that parental eating behaviors, lifestyle, and dietary attitudes are associated with children’s dietary habits.^23^^,^^24^ Our results are consistent with these earlier results, indicating that parents have a significant impact on their children’s dietary attitude and behaviors. Extending these results, we investigated more comprehensively the ways this may manifest. Because we evaluated the strength of the relationships quantitatively using BHPS as a lifestyle variable, we also found the dose-response relationships between low parental BHPS and lack of parental concern about diet or children eating breakfast alone, even after adjusting for family environment. The lower a parent scored with respect to healthy lifestyle factors, the less the parent seemed to prioritize or model positive dietary attitudes. If parents have little interest in healthy dietary habits, they may not know that eating healthy meals as a family is important for promoting their children’s health and protecting against unhealthy conditions, such as obesity and constipation.^5^^,^^6^ Therefore, we suggest that if parental lifestyle factors are undesirable, parents are more likely to let their children eat breakfast alone. Furthermore, children eating breakfast alone was also associated with the existence of other family members. We consider that if children live not only with parents but also with someone else, such as a grandfather or a grandmother, they could eat breakfast with their grandparents even if their parents are busy with work.

A previous study showed that mothers exert a stronger influence on children’s weight and were more concerned about their children’s eating behaviors than fathers.^25^ Using both paternal and maternal lifestyle variables in our analysis, we reveal that maternal BHPS is more strongly associated with parental dietary attitudes and dietary habits of children in comparison with paternal BHPS. This supports and extends the results of the previous study. In general, mothers spend more time with their children than do fathers and are more likely to prepare meals for the family.^26^ Thus, we may suppose that mothers are more likely to exert a greater degree of control over children’s dietary intake than fathers. We speculate that children’s dietary habits are influenced by the lifestyle factors of the person who mainly prepares family meals, regardless of whether that person is their father or their mother.

Through our analysis, we found that there are two types of dietary habits associated with lifestyle factors of both parents and children. As mentioned above, the dietary habits that were influenced by parental lifestyle factors were interest in Shokuiku, consideration of nutrient balance, and the person with whom children eat breakfast. Meanwhile, the dietary habits that were influenced by children’s lifestyle factors were the frequency of eating breakfast, vegetable intake, and snacking. Frequency of eating breakfast was associated with sleeping time and screen time; the shorter a child slept or the longer they used screens, the more likely the child was to skip breakfast. We suggest that if children sleep less and use screens for longer, they are more likely to wake up late and lack time to eat breakfast. Previous studies have shown that skipping breakfast is associated with childhood obesity, coming from a non-affluent family, and undesirable lifestyle factors.^3^^,^^13^^,^^27^ Although our results were partly inconsistent with these previous studies, this may be explained by the small sample size of individuals who reported skipping breakfast. In Japan, 10.6% of elementary school children do not eat breakfast every day, but only 6.5% of children in the present study reported skipping breakfast.^28^ Vegetable intake was associated with screen time and physical activity; more time spent with devices and lower physical activity were both associated with lower frequencies of eating vegetables. We speculate that children who exercise well are more likely to have hearty appetites than those who do not. Consequentially, there is a high possibility of these children frequently eating vegetables. Furthermore, the frequency of vegetable intake was also associated with indicators of family environment, such as family affluence and family members. We speculate that low-socioeconomic status (SES) families may not be able to afford to buy vegetables, meaning that their children are unable to eat fresh vegetables frequently. Although the relationship between the frequency of vegetable intake and family members is consistent with that found in a previous study, further study will be necessary to reveal its exact mechanism.^13^ Meanwhile, snacking was associated with screen time; the longer the screen time, the more likely children were to eat between meals. This result suggests that children are likely to eat snacks when they use various devices. Furthermore, we assume that such children were eating snacks frequently and so could not afford to eat vegetables and, consequently, their diets were more likely to be unhealthy.

Because of the range of associations uncovered in this study, different strategies may be necessary to address each dietary issue. Further studies would be required to identify other factors that may exert a direct impact on the frequency of eating breakfast, vegetable intake, and snacking.

Several limitations of the present study should be acknowledged. First, our study employed a cross-sectional design, and so we could not assess causal relationships. Although further longitudinal studies would be required to confirm the causalities, we may still reveal new findings, as detailed above. Second, our results may not be generalizable to a global population because we used only data collected from children at five elementary schools in Takaoka city, Toyama Prefecture, Japan. Third, our analyses were able to be adjusted only for family affluence as an SES variable. We could not include other SES variables, such as parental educational status and household income, in the questionnaire. Although our results could be overestimated owing to insufficient adjusting, that might be controlled to some extent by family affluence. Finally, every item in the analyzed questionnaire was self-reported by children and their parents. Although this could lead to misclassification of the variables, the validity of the instrument for assessing sleeping hours, physical activity, and BHPS has been confirmed in previous studies.^17^^,^^18^^,^^22^

In conclusion, dose-response relationships exist between undesirable parental lifestyle factors and lack of parental concern about dietary habits and undesirable breakfast habits of children, especially that of eating breakfast alone. Other dietary habits of children were more susceptible to the children’s own lifestyle factors than those of their parents. We suggest that nutrition education for parents may be especially important to improve parental dietary attitudes and to reduce the occurrence of children eating breakfast alone. However, specific, targeted nutrition education interventions may be required to appropriately address each dietary habit to improve the health outcomes of today’s children.
